# American Medical Association

**Published:** 1856-06

**Authors:** 


*American Medical Association.*—The American Medical Association has just completed its Ninth Annual Session, at Detroit. The session was one of great interest, and the members separated to carry to their several homes the most pleasant recollections of the hours spent together, and to spread, wherever they go, one loud and unanimous expression of admiration for the splendid hospitality with which they were entertained by the profession and citizens of Detroit.

The association met Tuesday morning, May 6th, at 11 o'clock, A. M., at Fireman's Hall. The meeting was called to order by the President, Dr. G. W. Wood, of Philadelphia, Dr. Wm. Brodie, of Detroit, being Secretary.

The association was welcomed to the state and city, by Dr. Z. Pitcher, of Detroit, in behalf of the committee of arrangements, in a happy speech.

After calling the roll of memberships, on motion of Dr. Thomson, of Delaware, a recess of fifteen minutes was taken to allow the delegates from the respective states to report one member from each state represented, as a committee to nominate officers for the ensuing year.

Twenty states and territories were already represented, and gave their votes in the above committee.

During the absence of the nominating committee, the committee of arrangements submitted a report, making out a plan for the hours of meeting, and for the exercises of the several days of the session; and also adversely to several propositions submitted by Prof. W. S. Davis, of Illinois, at the previous meeting, and designed to effect important changes in the manner of transacting the business of the association. The report was accepted.

The President announced the death of the eminent Dr. John C. Warren, of Boston, Mass., in that city, on Sunday morning.

Dr. Childs, of Mass., felt compelled to say a few words in this connection. He had been associated with the deceased for more than half a century, and should feel that he had been derelict of duty if he neglected to speak in his laudation. Dr. warren was the nephew of Joseph Warren, who fell gloriously at the battle of Bunker Hill. He was at the head of his profession in Massachusetts—had been President of the State Medical Society, and occupant of other elevated medical positions. His professional reputation was high, and his personal reputation spotless. His fame was not confined to Massachusetts. Though devoted to medical science, he was not limited to that alone, but paid attention to every branch of literature and art. If young members of the profession would be useful and eminent, they should follow the example of Dr. John C. Warren. To the older, the speaker would point out Dr. W.'s moral character as an exemplar. Such a life as his inevitably terminates in a death beatified by a surety of eternal happiness.

Dr. Gross, of Kentucky, made some remarks eulogistic of the deceased. He alluded to his high reputation—a reputation, he observed, not confined to America, but extending to every corner of the civilized world. Dr. Warren was the Nestor of American surgery. Dr. G. concluded by offering the following:

*Resolved*, That a committee of five be appointed to draft resolutions expressive of the feelings of this association at the loss of their late associate, Dr. John C. Warren.

The resolution was adopted, and the President appointed as such committee, Dr. Gross, of Kentucky, Dr. Childs, of Massachusetts, Dr. Wood, of New York, Dr. Pitcher, of Michigan, and Dr. Geddings, of South Carolina.

On motion, the association adjourned to 2, P. M.

### AFTERNOON SESSION.

Letters of invitation were received from the State Medical Society of Tennessee, and from lhe University of Nashville, inviting the association to hold its next annual session at Nashville, Tennessee, which were referred to the committee on nominations.

The committee on nominations unanimously nominated the following officers of the American Medical Association for the ensuing year:

*President*—Dr. Zina Pitcher, of Detroit.
*Vice-Presidents*—Drs. Thomas W. Blatchford, of New York; Wm. K. Bowling, of Tennessee; E. Geddings, of South Carolina; W. H. Brisbane, of Wisconsin.
*Secretaries*—Drs. Wm. Brodie, of Michigan; R. C. Foster, of Tennessee.
*Treasurer*—Dr. Casper Wisner, of Pennsylvania.

The report was accepted, and the nominations unanimously confirmed.

On motion of Dr. Atlee, of Pennsylvania, the President was requested to deliver his annual address.

We refrain from giving a report, because a sketch would not do justice to the admirable nature of the remarks, which should be read entire to be appreciated.

At the conclusion of the address, on motion of Dr. Atlee, of Pa.,

*Resolved*, That the thanks of the association be presented to our late President for the able and interesting parting address he has just delivered, and that he be requested to present to the committee of publication a copy, for preservation in our transactions.

On motion of Dr. Atlee, of Pa.,

*Resolved*, That a committee of three be appointed to inform the President and Vice-Presidents elect of their election, and conduct them to their seats.

The President appointed, as such committee, Drs. Atlee, of Pa., Reeves, of Ohio, and Sutton, of Kentucky.

Upon taking the chair, Dr. Pitcher said:

Although fully aware of my indebtedness, for this distinction, to your observance of a custom equivalent in force to positive law, of selecting your presiding officer, in each successive year, from the state in which the meeting of the association is held, I feel myself more honored by your partiality, than if I had received the same mark of respect from any other body of men known to the annals of our country.

This sentiment of regard for the body toward which I now hold, by this act of yours, so delicate and interesting a relation, has been inspired by a contemplation of the ideal of the physician, and strengthened by my growing acquaintance with the individuals which compose it.

Being unaccustomed to presiding in deliberative assemblies, I shall throw myself upon the indulgence of the association, and rely upon the kindness and intelligent coöperation of the individual members for assistance, in performing the duties of the chair.

Whilst thanking you most cordially for this expression of confidence, I can only assure you that such abilities as I possess shall be devoted to the prosperity of the association and the harmony of its proceedings.

On motion of Dr. Gunn, of Michigan,

*Resolved*, That the resolution passed at St. Louis, requiring a majority of the committee on publication to be appointed from residents of the place where the meeting is held, be repealed.

Dr. Phelps, of New York, offered the following:

*Whereas*, The pleasure and satisfaction of attending the deliberations of this association would be greatly enhanced, the duties of the secretaries and reporters facilitated, and order at the same time secured, by the observance of two things, to wit: first, that the audience be put in possession of the name and residence of the speaker; and, secondly, that they be enabled distinctly to hear what he has to say; therefore,

*Resolved*, That no one be permitted to address the association, except he shall have first given his name and residence, which shall be distinctly announced from the chair, and the member be required to go forward and speak from the stand, and not more than ten minutes at one time.

A motion to lay on the table was lost. The resolution was then adopted

At the request of Dr. Gross, of Ky., his report upon "The Causes that Retard Medical Education and Literature," was made the special order for Wednesday, at 10 o'clock.

The "Committee on Prize Essays," through Prof. Palmer, of Ill., reported that they had carefully examined the essays submitted to their inspection, and had judged one paper worthy of the prize. The title was, "An Essay on the Arterial Circulation."

Upon breaking the sealed envelop accompanying it, its author was announced as Dr. Henry Hartshorn, of Philadelphia.

Dr. Blatchford, of Troy, N. Y., from the committee on "Hydrophobia, and the Connection of the Season of the Year with its Prevalence," read a report thereon. The report abounds in many curious statistics and facts relative to this disease, and in conclusion submitted the following resolution, which was adopted:

*Resolved*, That the secretary transmit to the Governor of each state, a copy of the statistical part of this report, with the respectful request that he would bring the subject before the legislature of the state over which he presides, that in their wisdom they may devise and unite upon a plan by which the evil may be mitigated, if not removed.

The committee on nominations reported in favor of holding the next annual meeting of the association at Nashville, Tenn.

Dr. Gross, of Ky., moved to strike out "Nashville, Tenn.," and insert "Louisville, Ky." He thought Nashville at present difficult of access.

Dr. Geddings, of S. C., and Lindsley of Tenn., advocated the adoption of the report, and insisted that there was no difficulty in reaching Nashville, *provided* you did not pass *through* Louisville!

Dr. Gross finally withdrew his amendment, and the report was adopted with great unanimity.

Dr. Wistar, of Pa., from the committee on publication, read the annual report of the affairs of this department. The first copies of the transactions of 1855, were issued on the 10th of Nov. last, and an edition of 1100 copies printed, at an expense of \$1922 $\frac{70}{100}$. A more general circulation of the transactions was urged, and the coöperation of members in aid of such distribution was invoked.

The following resolution, passed at the last session, should be strictly enforced.

*Resolved,* That, hereafter, beginning with the session of 1856, no report, or other paper, shall be entitled to publication in the volume of the year in which it shall be presented to the association, unless it be placed in the hands of the committee of publication on or before June 1st.

The report further states that the number of volumes of transactions now remaining on hand, is as follows: of vol. I. 41, of vol. II. 9, of vol. III. 32, of vol. IV. 7, of vol. V. 316, of vol. VI. 66, of vol. VII. 120, of vol. VIII. 351; that some of the leading journals abroad have expressed a strong desire to complete their sets, and it rests with the association to determine whether the missing numbers shall be supplied; that, as only seven complete sets of the transactions are now in the possession of the association, the committee recommend that no copy of either of the eight volumes which is necessary to the complete sets now remaining shall be disposed of separately or with any number of volumes short of a complete set.

Dr. Atlee, of Pa., made some remarks upon the report, in the course of which he stated that the Smithsonian Institution had been offered as a permanent place of session for the association. He concluded by moving that the committee on publication preserve five complete sets of the proceedings. Carried.

On motion of Dr. Wood, of Philadelphia, the nomination of standing committees was referred to the committee on nominations.

The report of Dr. Breckenridge, of Ky., on Medical Literature, was made the special order next succeeding the report of Dr. Gross, which had been set down for Wednesday, at 10 o'clock, A. M.

Dr. Smith, of N. J., moved that that portion of the resolution requiring members, when speaking, to take the stand, be rescinded. Carried.

Dr. Atlee, of Pa., moved to refer the prize essay of Dr. Hartshorn, on Arterial Circulation, and the report of Dr. Blatchford on Hydrophobia, to the committee on publication. Carried.

Dr. Wistar, of Pa., the treasurer, read his annual report. It recommends that the treasurer be requested at an early date after the adjournment of the present meeting, to address a circular to each permanent member, announcing the abrogation of the resolution of 1854 — making a yearly subscription to the transactions obligatory — and the consequent restoration to membership of all those dropped from the published list of that year, — advertising, also, the practicability of procuring back numbers of the transactions, with information as to the cost at which the series of volumes may be rendered complete, or an entire set furnished by the association.

The receipts of the treasurer had been $3,584 26, and the expenditures $2,633 74, leaving a balance in the treasury of $950 52.

The report was accepted, and referred to the committee on publication.

Dr. McNulty, of the New York Academy of Medicine, offered a resolution that a committee of one from each state be appointed by the committee on nominations, to prepare and report to the association during the present session, an address to the people of the United States, setting forth the strong claims the medical profession have on their respect, gratitude and confidence.

By a member,— I move that the committee be named the Glorification Committee. (Laughter.)

Dr. McNulty explained the purpose for which he offered the resolution. Many people, he said, had a prejudice against the medical profession for holding to the dignities of their calling, and entertained the idea that the science of medicine was a collection of absurdities and superstitions. He wanted to show clearly that this is not the fact, aud, in this view, he thought the address proposed would have a beneficial effect.

Dr. Kittredge moved to amend the resolution by making it read, that every member of the association should take the stump and defend the cause of medicine, and their individual honor. (Laughter.)

After a few other remarks the resolution was withdrawn.

A gentleman, whose name we did not learn, stated that Dr. Wood, of New York, who was then in the meeting, had lately performed an operation in an extraordinary case, — removing a jaw-bone, — and moved that a time be appointed for the association to examine the part extirpated.

Dr. Wood said he had not with him the article spoken of by the preceding speaker, but would lay it on the desk of the President to-morrow morning.

The President read a communication from Dr. Stewart, chairman of the committee appointed last year to consider the subject of extending the lectures of each chair in medical schools over a period of two years, stating that the views of medical institutions had as yet been imperfectly ascertained, and asking a continuance of the committee. Granted.

The President read an invitation to the association to attend the session of the American Association for the Advancement of Science, at Albany, in August next,—at which time, also, the Dudley Observatory will be inaugurated, and an address delivered by Hon. Edward Everett. The invitation was accepted.

The association then adjourned to meet Wednesday morning at 9 o'clock.

On Tuesday evening the members of the association were received by Dr. H. P. Cobb, Dr. Morse Stewart, E. A. Brush and Albert Crane, Esqrs., at the latter gentleman's mansion. A new and agreeable feature in these hospitable entertainments was introduced, the presence of ladies.

### SECOND DAY.

The association was called to order by the President, at nine o'clock, and the minutes were read, corrected, and approved. The list of delegates who had registered their names since yesterday, was read.

A large number of letters were, as usual, received from the special committees, detailing reasons why they were unable to present their reports at this session, and asking an extension of time. The communications were severally referred to the committee on nominations.

The secretary announced that he had received the following resolution adopted at the last meeting of the New York State Medical Society:

*Resolved*, That the members of the American Medical Association be invited to attend the semi-centennial celebration of this society, which will occur on the first Tuesday of February, 1857.

The invitation was accepted.

The secretary read the following communication, dated April 7, 1856, from the Secretary of the Ohio State Medical Society:

Sir—At the annual meeting of this society, held in June last, at Zanesville, Ohio, the following resolutions were adopted, and I was directed to furnish you with a copy of the same:

*Resolved*, That the resolution offered by Dr. Grant (a member of this society, but not at this or at that time a practitioner of medicine, but a lawyer) at the last session of this society, viz: "That it is not derogatory to medical dignity, or inconsistent with medical honor, for medical gentlemen to take out a patent-right for medical, surgical or mechanical instruments," was offered at a time when many members had left for their homes, and is not, therefore, the sense of the society.

*Resolved*, That the said resolution is in direct opposition to the code of medical ethics adopted by this society; and therefore, be it further

*Resolved*, That said resolution, offered by Dr. Grant, and adopted by the society, be, and is hereby rescinded. (Applause.)

The communication was ordered to be placed upon the minutes.

The secretary read a communication from Dr. Hamilton, of Buffalo, N. Y., transmitting the second part of a report upon Deformities after Fracture and Dislocations, and asking for a correction of the minutes of last session in regard thereto. Dr. H. also asked that he be permitted to incorporate in a volume upon the subject he is preparing for publication, that portion of the report already published by the association.

On motion of Dr. Brodie, of Mich., the minutes were amended.

Dr. Atlee, of Pa., offered a resolution that the request of Dr. H. in regard to the publication of the report, be granted.

Dr. Lindsley, of Tenn., opposed the resolution. A similar request was denied at the session of the association held at St. Louis.

Dr. Palmer, of Ill., moved to refer the matter to a special committee. Carried.

The President appointed as such committee, Drs. Palmer of Ill., Atlee, of Pa., and Hills, of Ohio.

Dr. Gunn, of Mich., moved that those gentlemen from Canada, who are here by general invitation, be admitted in a body, and be requested to take seats on the platform during this morning's session. Carried.

In receiving them upon the platform, the President, Dr. Pitcher, said he was happy to be the instrument of celebrating the nuptials by which we effect a scientific reünion of the two members of the Anglo-Saxon race, which have so long been separated by the political relations having their origin in the separation of the American colonies from the English crown.

Dr. Hodder, in behalf of his Canadian brethren, thanked the association for the courtesy and kindness extended to them.

Dr. Sutton, of Ky., offered a resolution that 1,000 copies of the address of the late President, Dr. Wood, be published. Adopted.

On motion of Dr. J. B. Lindsley, of Tenn.,

*Resolved*, That a committee of three be appointed by the chair, to prepare a suitable minute in reference to the death of our late secretary, Dr. P. C. Gooch, of Richmond, Va., who fell a martyr while contending with the pestilence in Norfolk, in 1855.

The President appointed as such committee, Drs. Lindsley, of Tennessee, Thomson, of Del., and Mendenhall, of Ohio.

Dr. Gross, of Ky., from committee appointed the day previous, reported the following preamble and resolutions relative to the death of Dr. J. C. Warren, of Boston:

*Whereas*, It has pleased Almighty God to remove from the scene of his earthly labors our late fellow-member, Dr. John C. Warren, of Boston, formerly President of this association, and for many years Professor of Anatomy and Surgery in Harvard University.

*And whereas*, It is just and proper that, when a great and good man dies, his memory should be cherished by his fellow-citizens, and transmitted unimpaired to posterity for the encouragement of future ages; therefore,

*Resolved*, That this association has learned, with profound regret, the news of an event which has deprived the American medical profession of one of its oldest, most useful, and most illustrious members—American surgery one of its greatest ornaments—science one of its best friends—and humanity one of its noblest benefactors.

*Resolved*, That the life of Dr. John C. Warren affords an example of a man who, notwithstanding the possession of ample riches, devoted himself, heart and soul, for upwards of half a century, to the cultivation and advancement of his profession, and to the good of the human race.

*Resolved*, That this association deeply sympathizes with the family of Dr. Warren in their bereavement, and that the secretary be requested to transmit to them a copy of these proceedings.

The preamble and resolutions were adopted and referred to the committee on publications.

Dr. Gross, of Ky., read a report on "The Causes which Impede the Progress of American Medical Literature." In conclusion, he submitted the following resolutions:

*Resolved*, That this association earnestly and respectfully recommends: 1st. The universal adoption, whenever practicable, by our schools, of American works, as text-books for their pupils. 2d. The discontinuance of the practice of editing foreign writings. 3d. A more independent course of the medical periodical press toward foreign productions, and a more liberal one toward American; and, 4th. A better and more efficient employment of the facts which are continually furnished by our public institutions, for the elucidation of the nature of diseases and accidents, and, indirectly, for the formation of an original, a vigorous, and an independent national medical literature.

*Resolved*, That we venerate the writings of the great medical men, past and present, of our country, and that we consider them as an important element of our national medical literature.

*Resolved*, That we shall always hail with pleasure any useful or valuable work emanating from the European press, and that we shall always extend to them a cordial welcome, as books of reference, to acquaint us with the progress of legitimate medicine abroad, and to enlighten us in regard to any new facts of which they may be the repositories.

Dr. Phelps, of New York, moved that the report and resolutions, as a whole, be adopted.

At the suggestion of a member, the question was divided. The report was adopted.

Upon the reading of the first resolution, a member proposed to substitute "just" for "liberal," in line 8.

Dr. Gross accepted the amendment.

Dr. Palmer, of Ill., wished to understand the meaning of the word "practicable," as employed in the resolution, (line 3.) If it meant that an inferior work by an American author was to be used in our medical schools, in preference to a superior one, treating of the same subject, by a foreign author, he was decidedly opposed to the resolution. If, when the American work is equal or superior to the foreign one, it is to be used, then he had no objection. He alluded to works by eminent English and French authors.

Dr. Gross explained. One of the works alluded to by Dr. P. must of necessity be used in our medical institutions of learning, as there is no work by an American author on the same subject. Foreign works should be used as books of reference, and American books, "when practicable," as text-books.

Dr. Yandel, of Ky., moved that the resolutions be made the special order for Thursday morning. Lost.

Dr. Cobb, of N. Y., was opposed to the resolutions. If adopted and sent out to the world, they savor too much of know-nothingism to make them palatable. [Sensation.]

Dr. Leidy, of Pa., was in favor of leaving to teachers of medicine the selection of their own text-books.

Dr. Davis understood there was another report touching upon the subject —that upon "American Medical Literature," by Dr. Breckenridge, of Ky. He moved to lay the resolutions upon the table until that report was read Carried.

The secretary read a communication from Dr. P. A. Jewett, of Conn., chairman of the committee to procure memoirs of the eminent and worthy dead. Referred to committee on nominations.

Dr. Breckenridge, of Ky., read a report upon American Medical Literature. On motion of Dr. Hooker, it was accepted and referred to the committee on publications.

The association then adjourned to Thursday morning, at 9 o'clock.

On the afternoon of Wednesday, an excursion was given on board the steamer Western World, upon the river and lake, by the faculty of the city to their visiting medical brethren, and other invited guests. About two hundred ladies and six hundred gentlemen participated in it, and all expressed themselves highly gratified.

Dancing commenced shortly after leaving the city, and was continued without interruption until the return of the boat at half-past nine o'clock. A bountiful supper was prepared for the company in the after cabin.

Before separating for the night, a meeting of the members of the association was organized on the boat, by the election of Dr. N. B. Ives, of Conn., as chairman, and Dr. A. B. Palmer, of Ill., as secretary.

On motion, Dr. Atlee, of Pa., Dr. Conwell, of Tenn., and Dr. H. Monroe, of Me., were appointed a committee, who reported the following resolutions which were unanimously adopted:

*Resolved*, That the thanks of the members of the American Medical Association be presented to the Michigan Central Railroad Company, for their liberality in tendering to the committee of arrangements for our use, their magnificent floating palace, the steamboat Western World, for the excursion this afternoon and evening.

*Resolved*, That our thanks be presented to the captain and officers of said boat for their polite attentions, and especially to Mr. Wormer and the steward, for the careful preparation and beautiful arrangement of the luxurious viands provided by the committee for our entertainment.

*Resolved*, That the secretary be instructed to send a copy of the above resolutions to the President of the Michigan Central Railroad Company, J. W. Brooks, Esq., R. N. Rice, Esq., Superintendent, and Charles B. Swain, Esq., Agent.

THIRD DAY—MORNING SESSION.

The association was called to order by the President, at 9 o'clock.

The minutes were read, corrected, and approved.

A communication from Dr. Wroth, of Md., relative to a report upon the Medical Topography of the eastern shore of Maryland, and one from Dr. Thomson, of Ky., relative to a report on "Chloroform," were referred to the committee on nominations.

The secretary read a letter from E. S. Lesmoines, of St. Louis, enclosing an autograph letter from M. Dubois.

The secretary read a communication from J. C. Holmes, Esq., the secretary of the Michigan State Agricultural Society, presenting to the association twenty-five copies of the transactions of the society for 1853, and also the same number of the transactions for 1854.

Dr. Brodie, of Mich., moved that the thanks of the association be returned therefor, and that one copy be presented to each state representative. Carried.

On motion, Dr. Maggugin, of Iowa, was appointed a member from that state of the committee on nominations.

On motion of Dr. Atlee, of Pa.:

*Resolved*, That the President shall be authorized annually to appoint delegates to represent this association, at the meetings of the British Association, the American Medical Society of Paris, and such other scientific bodies in Europe as may be affiliated with us. Adopted.

Dr. Glück, of New York, offered the following:

*Whereas*, During the present year a medical congress is to be held in Europe; therefore

*Resolved*, That the American Medical Association send to that congress four delegates, representing the four sections of the Union.

Dr. Davis, of Ill., thought it might be necessary and proper to send a greater number than four. He moved to lay the resolution on the table. Carried.

Dr. Clendenin offered the following:

*Resolved*, That a committee of one be appointed, for a period of three years, with instructions to report progress at each annual meeting of this society, to investigate the etiology and pathology of epidemic cholera, and that said committee be allowed to add any other members to the same which they think may be necessary to further the objects of the appointment.

On motion, referred to the committee on nominations.

On motion of Dr. Mendenhall, of Ohio,

*Resolved*, That the secretary be instructed to strike the name of C. H. Cleveland from the list of permanent members of this association.

On motion of Dr. Atlee, of Pa.,

*Resolved*, That the name of James R. McClintock be stricken from the list of permanent members.

The expelled members were accused by the movers of the resolutions of having retrograded into quackery.

On motion of Dr. Bissell, of New York,

*Resolved*, That this association has learned with deep regret the death of one of its members, Dr. Theodore Romeyn Beck, of Albany, N. Y., whose whole life has been devoted to the attainment and promotion of medicine and general science, and that we do hereby express our high appreciation of the excellencies of his character, distinguished by its simplicity, integrity, and firmness of purpose, and by the extent and variety of his acquirements in medical as well as in almost every other department of science.

*Resolved*, That the above resolution be referred to the committee to procure memorials of the eminent and worthy dead, and that they be requested to procure a memoir of the late Dr. Beck, to be published in the transactions of the association.

Dr. Bloodgood, of Ill., offered the following:

*Resolved*, That the constitution of this association be so amended as that hereafter the President shall be elected by ballot, and shall not be resident of the state in which he is elected.

On motion of Dr. Watson, of N. Y., laid on the table.

*Dr. Wister*, of Pa., offered the following, which was adopted:

*Resolved*, That the invitation to gentlemen of the medical profession of Canada, extended to them by the American Medical Association at its session in Philadelphia, be renewed for the meeting at Nashville, Tenn.; and that this association may be safe from the introduction of unsuitable persons, it is recommended that gentlemen presenting themselves from the Province of Canada should be provided with a letter of introduction to this association from one of the following gentlemen: Drs. Tarquand, A. Scott, Woodstock, Canada; Drs. Hodder, Bethune, Richardson, Bonell, Haswell, Widmer, Beaumont, Herrick, of Toronto; Drs. O'Reilly, Craiggie, Duggan, of Hamilton; Dr. Sampson, of Kingston.

Dr. Phelps, of New York, offered the following:

*Whereas*, It has pleased an All Wise, but Inscrutible Providence, to visit the city of Norfolk, Va., and vicinity, with a desolating pestilence, equal, or surpassing, any recorded in ancient or modern times, and by which, in a few weeks, forty physicians, either residents or those from abroad, who had promptly rushed to the rescue, among the number of whom was our late secretary and associate, Dr. Gouch, of Richmond—had been swept away; therefore,

*Resolved*, That such an instance of signal and unflinching devotion to the cause of science and of humanity demands at the hands of this national association a passing expression of their high admiration of this, another memorable instance of the unparalleled sacrifices of the profession to the interests of the healing art and of the race.

*Resolved*, That this minute be incorporated in our transactions. Adopted.

On motion of Dr. Palmer, of Ill., Rt. Rev. Samuel A. McCoskry, Episcopal Bishop of this diocese, was invited to a seat upon the platform.

The like courtesy was extended to Dr. Mussey, formerly President of the association.

Dr. Stocker, of Pa., offered the following amendments to the constitution:

Amend article 3 so that it shall read: "Article 3. The regular meetings of the association shall be held annually, and commence on the first Tuesday of May. The association shall meet biennially in the city of ——. The place of meeting for the intermediate year shall be determined by a vote of the association."

Amend article 4 by providing for one permanent and two assistant secretaries, and also specifying the duties, &c., of each.

Laid on the table under the rule.

Dr. Dorsey, of Ohio, offered the following:

*Resolved,* That in May, 1858, and every third year thereafter, this association meet at Washington city, and that the present officers be requested to correspond with the Board of Managers of the Smithsonian Institute, in regard to furnishing necessary rooms for the keeping of the archives of the association.

Laid on the table under the rule.

On motion of Dr. Sheets,

*Resolved,* That it is derogatory to the dignity of the medical profession to notice the works of irregular practitioners in our medical periodicals.

Dr. Frost, of S. C., objected to the introduction of resolutions. He thought it irregular. Reports were the order.

Dr. Davis, of Ill., moved that reports be made the special order. Carried.

Dr. Watson, of N. Y., moved to reconsider the last vote in order to take up the resolutions attached to the report of Dr. Gross, of Ky., upon the "Causes which Retard American Medical Literature." Carried.

The resolutions were taken up. The question being upon their adoption:

Dr. Gross read extracts from his report, explained the intent of the resolutions, insisted upon their necessity, and advocated their adoption.

Dr. Davis, of Ill., was opposed to adopting any report. There were now before the association two reports, [the one by Dr. Gross, of Ky., and one by Dr. Breckenridge, of Ky.,] presenting directly adverse views. He thought both should be accepted and referred to the proper committee—nothing more.

Dr. Breckenridge, of Ky., said the point at issue is—whether the association will favor the sectionalism or the universality of medicine. If Dr. Gross' report and resolutions are adopted we decided in favor of the former.

Dr. Cobb, of N. Y., foresaw the difficulty that would arise in adopting Dr. Gross' report the day previous.

Dr. Watson, of N. Y., moved to reconsider the vote by which the report was adopted. Carried.

He then moved that the report be accepted. Carried.

On motion of Dr. Atlee, of Pa., the report and resolutions of Dr. Gross, and the report of Dr. Breckenridge, upon "American Medical Literature," were referred to the committee on publication.

Dr. Palmer, of Ill., from special committee to which was referred the communication of Dr. Hamilton, reported the following resolution, which was adopted:

*Resolved*, That leave be granted to Dr. F. H. Hamilton to make use of the materials of his report on "Deformities after Fractures," which is in course of publication by this association, in his anticipated work upon "Fractures and Dislocations."

Dr. A. B. Palmer, Professor in the Michigan University, from the committee on Plans of Organization for State and County Medical Societies, presented a lengthened and able report, containing numerous useful suggestions with outlines for the proper organization of local societies, and a series of resolutions in accordance with the views enforced in the report. Accepted, and referred to the committee on publication.

On motion, the resolutions were temporarily laid on the table for further action by the convention.

Dr. Davis, of Ill., chairman of special committee, reported on "The Changes in the Composition and Properties of the Milk of the Human Female, Produced by Menstruation and Pregnancy," in a paper containing numerous valuable details of much interest to the profession and the public, obtained by careful examination and comparison, and showing conclusively the ill-effects of lactation, especially during the latter of the periods referred to. Accepted, and referred to committee on publication.

Dr. Chas. G. Chandler, of Missouri, who was to report on "Malignant Periodic Fevers," submitted, as a substitute, through secretary Brodie, a paper on "Sulphate of Cinchona," which was received as a "voluntary contribution," and referred to a special committee.

Dr. J. M. Newman, of Buffalo, from committee on "The Sanitary Police of Cities," presented an elaborate report, embracing details of the various estimated causes of disease in cities, as compared with rural localities, together with numerous valuable statistics of mortality in the largest cities of Europe and the Union, of which the Doctor, at the request of the association, gave a brief verbal abstract. The report evidently embodies a vast mass of useful information, with deductions from it that city life is inimical to health and longevity, and arguments enforcing the urgent necessity for ameliorating the sanitary condition of the populous localities of cities and large towns. Of diseases arising from impure air, filth and insufficient ventilation, classed under the term "zymotic," the report stated that, in 1850, 40 per cent. of all the deaths in the United States were of that nature. The report also embodied details of the loss of life from cholera, small-pox, &c., giving startling expositions of danger from these sources, and recommends the enactment of laws for compulsory ventilation and cleanliness, as well as for vaccination, &c. Accepted and referred to committee on publication.

Adjourned to 2, P. M.

### AFTERNOON SESSION.

The asssociation met at 2 o'clock.

Dr. Frost, of Charleston, S. C., offered the following resolution, which was adopted:

*Resolved,* That the thanks of this association are due to the retiring officers for the zealous and efficient manner in which their duties have been performed; to our late President, for the courtesy and ability with which he has presided over our deliberations; to all the officers for their attention to the laborious duties of their stations—not excepting our committee on publication, to whom we must feel indebted for the satisfactory form in which the volume of the transactions appears.

Dr. A. J. Fuller, of Mo., chairman of the committee on the Best Treatment of Cholera Infantum, read a report thereon, in which he stated that the pathology of the disease was little understood, and that physicians should interchange views on the subject.

The report was accepted and referred to the committee on publication.

Dr. Green, of N. Y., chairman of the committee on the Use and Effects of Applications of Nitrate of Silver to the Throat, read a report thereon. He asserted that great benefits had been derived from topical medication in thoracic diseases,—tuberculosis, bronchitis, &c. The report was accepted and referred to the committee on publication.

Dr. Flint, of Louisville, chairman of the committee on the Best Mode of Rendering the Medical Patronage of the National Government Tributary to the Honor and Improvement of the Profession, read a report thereon. He denounced the granting of patents by the United States government to "quack medicines,"—stating, however, that it appears, from a letter written by the present Commissioner of Patents, that the practice of the office has been to discourage such a use of its functions, and that, during the past fifteen years, but four or five such patents have been granted, although from twenty to thirty applications therefor have been made per year. The credit of sanitary improvements, Dr. F. said, was not due to individuals, but to medical science. Such improvements are never discoveries or revelations, but inductions. The United States government should aid the great cause of medical science by making appropriations for the publication of the transactions of the National Association, and by paying prizes for the best essays on subjects selected by that association. The report was accepted and referred to the committee on publication.

The committee on nominations made the following report:

The nominating committee beg leave to make the following report:

For chairman of special committees for 1857:

Dr. E. R. Peaslee, of Brunswick, Me., on Inflammation, its Pathology and its Relation to the Recuperative Process.

Dr. J. C. Hutchinson, of Brooklyn, N. Y., and Charles E. Isaacs, of New York city, on the Anatomy and Histology of the Cervix Uteri.

Dr. J. Taylor Bradford, of Augusta, Ky., on the Treatment of Cholera.

Dr. Mark Stephenson, of N. Y., on the Treatment Best Adapted to Each Variety of Cataract, with the Method of Operation, Place of Election, Time, Age, &c.

Dr. J. W. Corson, of N. Y., on the Causes of the Impulse of the Heart, and the Agencies which Influence it in Health and Disease.

Dr. D. Meredith Reese, of N. Y., on the Causes of Infant Mortality in Large Cities, the Source of its Increase, and the Means for its Diminution.

Dr. J. Foster Jenkins, of Yonkers, N. Y., on Spontaneous Umbilical Hæmorrhage of the Newly Born.

Dr. Henry Carpenter, of Lancaster, Pa., on the Use of Instruments in Obstetrical Practice.

Dr. Alexander J. Semmes, of Washington, D. C., on the Measures to be Adopted to Remedy the Evils Existing in the Present Mode of Holding Coroner's Inquests.

Dr. J. Marion Sims, of New York city, on the Treatment of the Results of Obstructed Labor.

Dr. J. B. Flint, of Louisville, Ky., on the True Position and Value of Operative Surgery as a Therapeutic Agent.

Dr. G. Volney Dorsey, of Piqua, Ohio, on the Causes and Cure of Indigestion, especially in Relation to the Therapeutical Indications to be derived from the Chemical Composition of the Deposits in the Urine.

Dr. C. B. Coventry, of Utica, N. Y., on the Medical Jurisprudence of Insanity, and the Testimony of Skilled Witnesses in Courts of Justice.

Dr. Joseph Leidy, of Philadelphia, Pa., on Human, Animal, and Vegetable Parasites.

Dr. M. D. Darnall, of Bainbridge, Ind., on the Value of a Strict Attention to Position in the Treatment of Diseases of the Abdomen.

Dr. George Sutton, of Aurora, Ind., on Milk Sickness.

Dr. Clark J. Pease, of Janesville, Wis., on the Blending and Conversion of the Types of Fever.

Dr. B. S. Woodsworth, of Fort Wayne, Ind., on the Best Substitute for Cinchona and its Preparations in the Treatment of Intermittent Fever and Malarious Neuralgia.

Dr. Franklin Hinkle, of Marietta, Pa., on the Use of Cinchona in Malarious Diseases.

Dr. Henry V. Campbell, of Augusta, Ga., on the Nervous System in Febrile Diseases.

Dr. John Neill, of Philadelphia, Penn., on the Laws Governing the Deposit of Bone.

Dr. John W. Greene, of New York city, on the Intimate Effects of Certain Toxicological Agents in the Animal Tissues and Fluids.

Dr. George Suckley, U. S. A., on the Medical Topography and Fauna of Washington Territory.

Dr. James Cooper, of Hoboken, N. J., on the Flora of Washington and Oregon Territories.

Dr. Chas. E. Isaacs, of N. Y., on the Intimate Structure and the Pathology of the Kidney.

Dr. Israel Moses, of New York city, on the Diseases Incidental to Emigrants from Temperate Climates in their Transition through Central America.

Dr. T. W. Gordon, of Georgetown, Brown county, Ohio, on the Etiology and Pathology of Epidemic Cholera, to be continued three years, and with power to add any other members.

Dr. H. A. Johnson, of Chicago, on the Excretions as an Index to the Organic Changes going on in the System.

Dr. D. D. Thomson, of Louisville, on the Remedial Effects of Chloroform.

Standing Committees. — *Committee on Publication.* — Drs. Francis G. Smith, of Pa., Chairman; Caspar Wister, of Pa.; Samuel L. Hollingsworth, of Pa.; Samuel Lewis, of Pa.; H. F. Askew, of Del.; Wm. Brodie, of Mich.; R. C. Foster, of Tenn.

*Committee on Prize Essays.* — Drs. Wm. K. Bowling, of Tennessee, Chairman; E. B. Haskins, of Tenn.; Thomas Lipscomb, of Tenn.; A. H. Buchanan, of Tenn.; B. W. Avent, of Tenn.; W. A. Cheatham, of Tenn.; Paul F. Eve, of Tenn.

*Committee of Arrangements.* — Drs. C. K. Winston, of Tenn., Chairman; Ira Conwell, of Tenn.; William D. Haggart, of Tenn.; J. L. C. Johnson, of Tenn.; F. A. Ramsay, of Tenn.; Geo. Grant, of Tenn.; J. B. Lindsley, of Tenn.

To fill vacancies in the committee on medical topography and epidemics:

*New Hampshire.* — Dr. V. P. Fitch, of Amherst.

*California.* — Dr. Robert Murray, of Fort Miller.

To fill vacancies in the committee upon a uniform system of registration of marriages, births, and deaths:

*Vermont.* — Dr. Adrian T. Woodward, of Castleton.

*Connecticut.* — Dr. Wm. B. Casey, of Middletown.

*Virginia.* — Dr. R. W. Haxall, of Richmond.

*California.* — Dr. Arthur R. Stout, of San Francisco.

They recommend the continuance of the "Committee to Procure Memorials of the Eminent and Worthy Dead," and that the report, as far as prepared, be referred to the committee on publication.

Standing Committees. — *On Medical Education.* — Drs. E. Geddings, of S. C., chairman; C. W. Le Boutillier, of Minnesota; G. F. Mitchell, of Ohio; S. W. Clanton, of Ala.; S. W. Butler, of N. J.

*On Medical Literature.* — Drs. R. Hills, of Ohio, chairman; D. W. Yandell, of Ky.; R. R. Porter, of Del.; H. A. Johnson, of Ill.; Charles E. Swan, of Maine.

The President stated that Dr. Anderson, of Ala., chairman of committee on medical education, had sent in his report. It was accepted and referred to the committee on publication.

A report from Dr. Worth, of Maryland, on the Medical Topography and Epidemics of the Eastern Shore of Maryland, was accepted and referred to the committee on publication.

A report from Dr. Cain, of S. C., on the epidemic of yellow fever in Charleston in 1854, was accepted and referred to the committee on publication.

A report from Dr. Fenner, of La., on the Medical Topography and Epidemics of Louisiana, was accepted and referred to the commitee on publication.

Secretary Brodie stated that he had received a letter from Dr. Dillard, U. S. N., appointed on the committee on medical topography and epidemics, saying that he could not act, in consequence of having received no appointment as delegate to the association from the Surgeon General.

Dr. Gunn, of Michigan, said three communications had been handed to the committee of arrangements by the secretaries, which they (the committee) had not time to examine. He asked that a special committee be appointed to report on volunteer communications.

Dr. Palmer, of Illinois, offered the following, which was adopted:

*Resolved*, That the volunteer communications in the hands of the committee of arrangements be referred, with all other such communications, to a special committee to be appointed by the chair, residing at the place of publication of the transactions ; and if, in their judgment, the papers are worthy, they be referred by them to the committee on publication, to go into the transactions of the association.

The President appointed as such committee, Drs. A. Stilli, S. Jackson, and F. J. B. Biddle.

The authors and titles of the volunteer communications were announced by Secretary Brodie as follows:

By Dr. C. J. Chandler, of Rocheport, Mo., on Sulph. Cinchona in Periodic Diseases.
By Dr. Isidor Gluck, of New York, on Formation of Gun Shot Wounds, &c.
By Dr. G. P. Flachenberg, on an Improved Method of Applying Compression to the Scrotum.

A member of the committee on a uniform system of registration of marriages, births, and deaths, stated that they were unable to make a report at present, in consequence of the death of their chairman, Dr. Wilson, of Conn.

The committee on medical literature, for 1855, was continued for another year.

Dr. Neill, of Philadelphia, offered a resolution that no medical preparation, account of surgical operation, or anything else designed or calculated to give notoriety to an individual, be laid before the association, until reported upon by a special committee.

Dr. Wood, of N. Y., presumed that this resolution was aimed at him. He had come here with the description of a disease never before described by surgeons — (phosphorous disease of jaw-bone.) He had felt great delicacy in inviting the attention of the association to the subject; and it was not until after consultation with many of the most prominent members of the body, that he had permitted a friend to do so. As for the charge of seeking notoriety, he denied it *in toto*. He had aimed at no such purpose, and he felt wounded at the tone of the resolution.

Much applause followed the conclusion of Dr. W.'s remarks.

Dr. Neill disclaimed the intention of personal allusion in the resolution he had offered. That resolution embodied a principle which never should be violated. Dr. Wood's reputation, or notoriety, might not be enhanced by the action under reference, but the privilege of similarly proceeding might be abused by other persons hereafter.

Dr. N.'s remarks were received with applause.

Dr. Wood said he had heard beforehand that such a resolution was to be offered. He had been told that his jaw was to be pulled; that the operation would give him too much notoriety; and it was not the resolution itself he cared so much about, as the outside talk. He expressed a desire that the motion of Dr. Gross, of Kentucky, inviting the association to examine his (Dr. W.'s) surgical specimen, would be stricken from the minutes.

Dr. Thomson, of Delaware, made some humorous remarks. He hoped that New York would hold her jaw, and Philadelphia not stick in hers. [Great laughter.] He trusted that Dr. Neill would withdraw his resolution, and that Dr. Gross' motion would be stricken from the minutes. If these were done, he would see that all was made right between the opposing gentlemen before they reached home. [Laughter.]

Dr. Gross moved to strike his motion referred to from the minutes — for the purpose, he said, of removing the-bone of contention. [Great laughter.]

Dr. Neill withdrew his resolution, and Dr. Gross' motion was stricken from the minutes.

Dr. Gross, of Louisville, tendered, in behalf of the medical profession and the citizens of Louisville, an invitation to the association to meet in that city in May, 1858. Placed on file.

Dr. Dorsey, of Ohio, offered the following resolution, which was adopted:

*Resolved*, by the American Medical Association, That the committee of the etiology and pathology of cholera be instructed to memorialize the Congress of the United States, requesting that honorable body to grant every necessary assistance which can or will promote the objects for which the committee has been appointed.

Secretary Brodie read a communication from the Royal Medical and Chirurgical Society of England, thanking the American Medical Association for their present of the eighth volume of their transactions. Ordered placed on file.

Dr. Wister, of Pa., offered the following, which was adopted:

*Resolved*, That a committee of three be appointed by the President, to correspond with the proper officer of the Smithsonian Institute, inquiring into the possibility of procuring a chamber in that institution for the uses of this association.

The President appointed as such committee, Drs. Wister, of Pa., Hale, of Washington, and J. Neill, of Pa.

Dr. Phelps, of N. Y., offered the following, which were adopted.

*Resolved*, That the thanks of this association are due, and are hereby tendered, to the Fire Department of the city of Detroit, for the gratuitous use of their large and commodious hall, so amply furnishing to us accommodation for the convenient transaction of business.

*Resolved*, That the urbane deportment and elegant hospitalities of the profession and of private individuals, as well as the polite attentions of citizens generally, demand of this association a high appreciation of the cultivated manners of this city of the West, and which has tended greatly to enhance the pleasure of the session here of the delegates from abroad.

The association adjourned till Friday morning, nine o'clock.

LAST DAY.

The Association was called to order by the President at nine o'clock.

The minutes were read, corrected, and approved.

Dr. Palmer, of Ill., moved that Dr. Coolidge, U. S. A., be substituted in place of Dr. Finley, U. S. A., as a member of the committee on medical topography and epidemics. Dr. P. said he made the motion by request. Carried.

Dr. Atlee, of Pa., offered the following, which was adopted:

*Resolved*, That all voluntary communications hereafter presented to the association shall be referred to a special committee, to be appointed by the president on the first day of each annual meeting, whose duty it shall be to examine such communications and report upon the propriety of their presentation and reference to the committee of publication.

Dr. Lindsley, of Tenn., from the special committee appointed the day previous, reported the following preamble and resolutions :

*Whereas*, The exhibition of high courage and of self-sacrificing devotion to the good of others is ever honorable to a profession by whose members it is manifested, and worthy of their remembrance and emulation; therefore,

*Resolved*, That in the death of P. Claiborne Gooch, of Richmond, Va., who nobly volunteered his services during the pestilence at Norfolk, we recognise a loss to this association, the profession, and the country. His arduous and successful labors as secretary of the meetings at Charleston and Richmond, merited the regard of this association. The zeal, ability, and industry manifested by him as founder and editor of the *Stethoscope* — the first medical periodical established in the State of Virginia — showed his devotion to the cause of medical progress and activity; and the manner of his death gave signal evidence that he was one of whom his country might well be proud.

*Resolved*, That a copy of these resolutions be transmitted by the secretary to the relatives of the late Dr. Gooch.

The resolutions were adopted, and had the usual reference.

On motion of Dr. Palmer, of Illinois,

*Resolved*, That the committee on registration have leave now to present a partial report, which is hereby referred to the committee on publication.

Dr. Denton, of Mich., offered the following:

*Resolved*, That a committee of three shall be appointed, whose duty it shall be to enlist some enterprising publisher to aid in collecting and arranging material for an American Medical Directory.

On motion of Dr. Watson, of N. Y., laid on the table.

Dr. Leide, of Pa, offered the following:

*Whereas*, It is the object of this association, in the award of prizes for communications on subjects appertaining to medical science, to encourage the progress of the latter; and as this result cannot be better attained than through original investigation and discovery —

*Resolved*, That hereafter an annual prize of —— dollars be awarded for the best memoir or essay founded on original investigations of the author; and in case of no memoir or essay being presented worthy of such award, the prize money to be appropriated towards the expense of publishing and illustrating such memoirs as may be subsequently deemed worthy of an award.

The resolution, together with the suggestions of the committee on prize essays, as to whether any means can be devised to cause an increase of the number of essays presented, were referred to a special committee, consisting of Drs. Leide, Wood, and C. D. Meigs, of Pa.

Proposed amendments to the constitution being in order, Dr. Smith moved that the proposition to amend by providing that "any member who omits to pay for the published transactions for three successive years shall be considered as withdrawn," be laid upon the table until the next annual meeting of the association. Carried.

The Secretary read an invitation to the association to attend the next annual meeting of the Massachusetts State Medical Association. Accepted.

Dr. R. K. Smith offered the following:

*Resolved,* That a special committee be appointed to report for the next meeting of the American Medical Association a classification of those diseases which involve a derangement of the mental manifestations.

Adopted, and Dr. Smith appointed chairman of said committee, with power to choose his associates.

On motion of Dr. Atlee,

*Resolved,* That the committee on publication be requested to transmit annually to the Epidemiological Society of London a copy of our transactions.

On motion of Mr. Gunn, of Michigan,

*Resolved,* That any new medical institution not heretofore represented in this body be requested to transmit to the secretary, with the credentials of its delegates, evidence of its existence, capacity, and good standing.

Dr. Phelps, of N. Y., offered a preamble and resolutions relative to the relation existing between medicine and religion. Laid on the table.

Dr. McGuggin offered the following:

*Resolved,* That a special committee be appointed to report on the subject of "Stomaidis Materna."

Adopted, and Dr. McGuggin appointed chairman of such committee.

On motion of Dr. Bailey, of Illinois, Dr. Davis, of Chicago, was requested to continue his observations on the changes produced in the composition and qualities of milk by pregnancy and menstruation; also, the best substitute for the mother's milk when weaning becomes necessary; and report at the next meeting of the association.

A report from the committee on railroads, &c., was read, and the same committee continued to next meeting.

On motion of Dr. Smith, of N. J., the resolutions of Dr. Palmer, offered the day previous, were taken from the table and referred to the committee on publication.

On motion of Dr. Atlee, of Pa., the thanks of the association were returned to those railroads that had evinced a liberality in conveying delegates to and from the association.

On motion of Dr. Palmer, of Illinois,

*Resolved*, That the thanks of the association be presented to the press of the city of Detroit, who have taken so much interest in reporting the proceedings of this meeting.

The association then adjourned to meet in Nashville, Tenn., in 1857.

About three hundred members were in attendance during the session.

